# PD-1/PD-L1 inhibitors in the treatment of pMMR mCRC: a meta-analysis based on randomized controlled trials

**DOI:** 10.3389/fonc.2026.1969514

**Published:** 2026-10-21

**Authors:** Sumin Lv, Chaoyue Ma, Zeqi Tang, Xinyan Zhang, Wen Shi, Shiyan Zhang, Minyuan Ni, Lihu Gu, Bangsheng Chen

**Affiliations:** 1 Department of General Surgery, Ningbo No.2 Hospital, Wenzhou Medical University, Ningbo, Zhejiang, China; 2 The Fifth Clinical Medical College of Zhejiang Chinese Medical University, Hangzhou, Zhejiang, China; 3 The Second Clinical Medical College of Zhejiang Chinese Medical University, Hangzhou, Zhejiang, China; 4 Emergency Medical Center, Ningbo Hospital of Integrated Traditional Chinese and Western Medicine, Ningbo, Zhejiang, China

**Keywords:** chemotherapy, MCRC, meta-analysis, PD-1/PD-L1 inhibitors, PMMR, prognosis

## Abstract

**Background:**

The clinical efficacy of PD-1/PD-L1 inhibitors combined with chemotherapy (± targeted agents) in patients with proficient mismatch repair (pMMR) metastatic colorectal cancer (mCRC) remains unclear. This study systematically evaluated the efficacy of this regimen.

**Methods:**

We searched PubMed, Web of Science, Embase, the Cochrane Library, and ClinicalTrials.gov for relevant randomized controlled trials (RCTs) published up to May 2026. The primary outcomes were progression-free survival (PFS) and overall survival (OS); effect sizes were estimated as hazard ratios (HRs) and 95% confidence intervals (95%CIs).

**Results:**

Six RCTs comprising 1,165 participants were included. The addition of PD-1/PD-L1 inhibitors to chemotherapy, with or without anti-VEGF agents, was associated with prolonged PFS (HR = 0.84, 95%CI [0.73, 0.95], P = 0.007) and OS (HR = 0.83, 95%CI [0.73, 0.94], P = 0.004). Subgroup analyses for PFS preliminarily suggested greater benefit in male patients (HR = 0.77, 95%CI [0.64, 0.94], P = 0.010), those with an Eastern Cooperative Oncology Group (ECOG) performance status of 0 (HR = 0.73, 95%CI [0.56, 0.95], P = 0.018), those with liver metastases (HR = 0.82, 95%CI [0.68, 0.98], P = 0.027), those without prior adjuvant therapy (HR = 0.81, 95%CI [0.67, 0.98], P = 0.030), those with a PD-L1 combined positive score (CPS) < 1 (HR = 0.53, 95%CI [0.30, 0.95], P = 0.032), those with synchronous metastases (HR = 0.79, 95%CI [0.63, 0.99], P = 0.037), and those with Kirsten rat sarcoma viral oncogene homolog (KRAS) mutations (HR = 0.49, 95%CI [0.30, 0.80], P=0.005). These subgroup findings are exploratory, and interaction tests were not performed.

**Conclusions:**

PD-1/PD-L1 inhibitors combined with chemotherapy (with or without targeted agents) may improve the prognosis of patients with pMMR mCRC and represent a potential first-line treatment option.

**Systematic review registration:**

https://www.crd.york.ac.uk/PROSPERO/, identifier CRD420261330186.

## 1 Introduction

Colorectal cancer (CRC) is one of the most common malignancies worldwide in terms of both incidence and mortality. In 2022, there were approximately 2,000,000 new cases of CRC globally, accounting for 9.6% of all cancers, ranking third only to lung cancer and female breast cancer. The number of deaths reached approximately 900,000, representing 9.3% of all cancer mortality, ranking second only to lung cancer (1). Owing to the insidious onset of CRC symptoms, nearly one-third (33.2%) of patients have already developed metastatic disease at initial diagnosis (2), thereby precluding curative surgical resection and underscoring the urgent need for effective systemic therapies in this population.

Treatment strategies for metastatic CRC (mCRC) must be tailored according to mismatch repair (MMR) status (3). Among CRC patients, only approximately 15% are mismatch repair-deficient (dMMR) or have microsatellite instability-high (MSI-H) (4). For this subset, first-line PD-1/PD-L1 inhibitor monotherapy or dual immunotherapy has shown marked efficacy and favorable survival outcomes (5). However, approximately 85% of CRCs are of the proficient mismatch repair (pMMR) subtype; pMMR mCRC is generally considered an immunologically “cold” tumor in which PD-1/PD-L1 monotherapy achieves an objective response rate (ORR) of less than 5% (6, 7). Systemic therapy for patients with pMMR mCRC still predominantly relies on chemotherapy combined with targeted agents, primarily directed against vascular endothelial growth factor (VEGF) and epidermal growth factor receptor (EGFR) (3). Consequently, PD-1/PD-L1 inhibitors have long been considered ineffective for pMMR mCRC, and immunotherapy research in this setting has progressed slowly owing to disappointing early-phase results.

Previous studies have suggested that the mechanisms underlying the “cold” tumor phenotype of pMMR tumors involve multiple levels (8). First, pMMR tumors have low tumor mutational burden (TMB) and insufficient neoantigens (9), making it difficult to activate dendritic cells (DCs) and T cells (10, 11). Second, infiltration by regulatory T cells (Tregs), myeloid-derived suppressor cells (MDSCs), tumor-associated macrophages (TAMs), and cancer-associated fibroblasts (CAFs) creates an immunosuppressive microenvironment (12, 13). Third, impaired antigen presentation and upregulated co-inhibitory ligands lead to T-cell exhaustion (8, 14–17); moreover, Wnt/β-catenin activation upregulates activating transcription factor 4 (ATF4), inhibits conventional type 1 dendritic cells (cDC1s), and weakens cross-presentation and CD8+ T-cell priming, thereby contributing to the “cold” phenotype (18–21).

However, recent Food and Drug Administration findings indicate that TMB cannot serve as a predictive biomarker of response to PD-1/PD-L1 immunotherapy (22), suggesting that “cold” tumors may be driven primarily by immunosuppression rather than by low TMB alone. Importantly, systemic therapy has the potential to disrupt the immunosuppressive microenvironment and convert “cold” tumors into “hot” tumors, an effect that operates at two complementary levels. At the cellular level, chemotherapy and anti-VEGF therapy reduce the infiltration of immunosuppressive cells: 5-fluorouracil depletes MDSCs and enhances CD8+ T-cell activity (23); anti-VEGF therapy reduces Tregs and MDSCs, inhibits M2-like TAMs, and promotes their polarization toward M1-like TAMs (24–26). At the metabolic level, anti-VEGF therapy improves oxygen supply, restores prolyl hydroxylase (PHD) activity, promotes hypoxia-inducible factor (HIF)-α degradation, reduces HIF-1α/2α activity, and thereby attenuates downstream immune evasion and drug resistance (27–31). By enhancing antigen presentation, promoting CD8+ T-cell infiltration, and relieving immunosuppression, combination therapy may enable PD-1/PD-L1 inhibitors to restore the function of exhausted T cells (32, 33). Chemotherapy can also augment the innate arm of antitumor immunity: it enhances natural killer (NK) cell function and increases tumor-cell expression of NKG2D ligands, DNAM-1 ligands, and TRAIL receptors (34–36), rendering tumor cells more susceptible to NK cell recognition and clearance and increasing the sensitivity of cancer stem cells to NK cells (37). Together, these mechanisms—operating at both cellular and metabolic levels and engaging both adaptive and innate immunity—provide the background for understanding combination therapies.

Recently, various combination strategies incorporating immunotherapy have been explored for pMMR mCRC, including anti-VEGF plus immune checkpoint inhibitors (ICIs), EGFR-targeted therapy plus ICIs, chemotherapy plus ICIs, radiotherapy or ablation plus ICIs, and dual ICIs (38). A previous systematic review and meta-analysis of four randomized controlled trials (RCTs) demonstrated that PD-1/PD-L1 inhibitors combined with chemotherapy yielded a preliminary improvement in progression-free survival (PFS) (hazard ratio (HR)=0.82, 95% confidence interval (95%CI) [0.70, 0.97], P = 0.02) but did not demonstrate a significant overall survival (OS) benefit (HR = 0.85, 95%CI [0.65, 1.11]) (39). Given the availability of updated data from an increasing number of RCTs, an updated meta-analysis is warranted to systematically evaluate the efficacy of PD-1/PD-L1 inhibitors combined with chemotherapy, with or without targeted therapy, in pMMR mCRC and to identify factors influencing treatment outcomes through subgroup analyses, thereby providing evidence-based support for clinical decision-making.

## 2 Methods

This systematic review and meta-analysis was conducted in accordance with the PRISMA guidelines (40). The study was registered with the prospective register of systematic reviews, PROSPERO (ID: CRD420261330186).

### 2.1 Search strategy

The following databases were systematically searched: PubMed, Web of Science, Embase, the Cochrane Library, and ClinicalTrials.gov. The search covered the period from database inception to May 2026 to identify relevant RCTs investigating PD-1/PD-L1 inhibitors for the treatment of mCRC. A combination of MeSH terms and free-text keywords was employed, with tailored search strategies developed for each database. The search strategy used the following keywords: (“Colorectal Neoplasms”[Mesh]) OR (Colorectal Tumor) OR (Colorectal Cancer) OR (Colorectal Carcinoma) AND (“Immunotherapy”[Mesh]) OR (Programmed Cell Death Protein 1 Inhibitor) OR (Programmed Death Ligand 1 Inhibitors) OR (Atezolizumab) OR (Nivolumab) OR (Pembrolizumab) OR (Ipilimumab) OR (Serplulimab) OR (Tislelizumab) AND (“Drug Therapy”[Mesh]) OR (Chemotherapy) OR (Pharmacotherapy) OR (Therapy, Drug). The reference lists of the included articles were manually searched to avoid potential omissions. All retrieved records were imported into EndNote. After automated deduplication, the remaining records were manually screened based on titles, abstracts, and full texts. The screening process was conducted independently by two reviewers. Any disagreements were resolved through discussion and consensus between the two reviewers or by adjudication of a third party.

### 2.2 Inclusion criteria (PICOS criteria)

1. Study participants were patients suffering from pMMR mCRC;
2. The experimental group received PD-1/PD-L1 inhibitors combined with chemotherapy, with or without targeted therapy;
3. The control group received chemotherapy, with or without targeted therapy;
4. The primary outcomes were PFS and/or OS;
5. The study design was an RCT.

### 2.3 Exclusion criteria

1. Studies were excluded if they had incomplete data, if full texts were not accessible, or if the data could not be extracted for statistical analysis;
2. Non-English articles;
3. When a study had updated publications, the article with the most comprehensive or most recent data was included.

### 2.4 Data extraction

Two reviewers independently extracted relevant data from each included study using a pre-designed data extraction form. The extracted content included: basic study information (authors, publication year, trial registration number, country, clinical trial phase, study recruitment period), study design (treatment regimen, sample size, blinding method, line of therapy), and outcomes (data related to OS and PFS).

Risk assessment of bias. The risk of bias for each included RCT was independently assessed by two reviewers using the Cochrane Risk of Bias tool. Disagreements were resolved through discussion or arbitration by a third reviewer. This tool evaluates potential bias across seven domains: random sequence generation, allocation concealment, blinding of participants and personnel, blinding of outcome assessment, incomplete outcome data, selective reporting, and other bias. Each domain was categorized as having a low, high, or unclear risk of bias.

### 2.5 Statistical analysis

The data analysis was performed using Stata software (version 18.0) and Review Manager software (version 5.3). Treatment efficacy was evaluated using pooled HRs with 95% CIs for PFS and OS. An HR < 1 favored the experimental group, whereas an HR > 1 favored the control group. Given the potential clinical heterogeneity among the included studies, including differences in study design, population characteristics, and outcome measures, a random-effects model was prespecified for data synthesis to enhance the reliability of the results. Statistical heterogeneity was assessed using the chi-square test and the I² statistic. An I² ≥ 50% and a p ≤ 0.05 were considered indicative of significant statistical heterogeneity, while an I² < 50% and a p > 0.05 indicated low heterogeneity. Publication bias was assessed using Egger’s test. To evaluate the robustness of the results, a sensitivity analysis was performed by sequentially excluding one study at a time. To explore potential sources of heterogeneity, subgroup analyses were performed according to a prespecified plan, with grouping factors including age, sex, and Eastern Cooperative Oncology Group (ECOG). All statistical tests were two-tailed, and p < 0.05 was considered statistically significant.

The certainty of evidence for PFS and OS was assessed using the Grading of Recommendations Assessment, Development and Evaluation (GRADE) approach. Since all included studies were RCTs, the initial certainty rating for each outcome was high; it was then downgraded by 1 or 2 levels according to the five domains of risk of bias, inconsistency, indirectness, imprecision, and publication bias, and the final certainty of evidence was rated as high, moderate, low, or very low.

## 3 Results

### 3.1 Study search and screening

A preliminary literature search was conducted across the databases, yielding 6,269 records, with no additional articles identified through other sources. After automated deduplication using EndNote software, 5,741 records remained. Following screening by title and abstract, the majority of articles were excluded, leaving 36 articles for full-text review. Of these, 30 studies were excluded for not meeting the inclusion criteria (e.g., non-RCTs, unavailable data, protocol non-compliances, or updated studies), ultimately leaving 6 articles for inclusion (41–46). The detailed screening process and results are illustrated in **Figure 1**.

**Figure 1:**
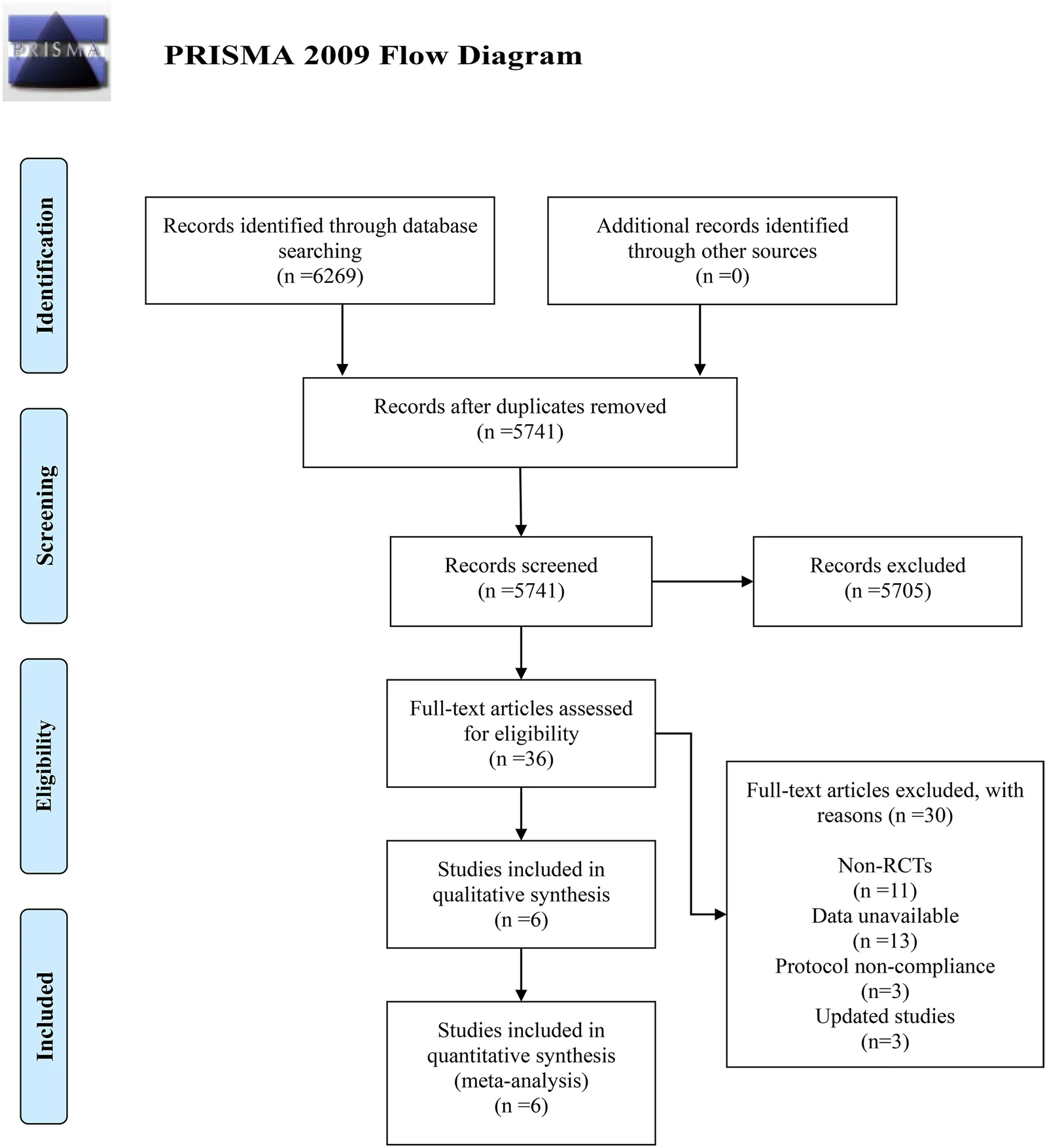
PRISMA flow diagram.

### 3.2 Characteristics of included studies

This meta-analysis included six RCTs involving 1,165 patients with mCRC, with 525 patients in the experimental group and 640 patients in the control group. Of these patients, 88.5% had pMMR tumors. These studies were published between 2022 and 2024, with enrollment periods ranging from 2015 to 2022. Five studies were phase II and one was a phase II/III trial; five were open-label and one was double-blind; five were conducted as first-line therapy and one was second-line or later; two were multicenter, and the remaining four were conducted in Italy, the United States, Norway, and China, respectively. In five studies, the experimental group received PD-1/PD-L1 inhibitors combined with chemotherapy and VEGF-targeted agents, whereas in one study the experimental group received PD-1/PD-L1 inhibitors combined with chemotherapy alone. The primary endpoint in all six studies was PFS. One of these studies did not report mature overall PFS or OS data and provided only age subgroup data. Detailed characteristics of the included studies are presented in **Table 1** and **Supplementary Table 1**.

**Table 1:** Basic characteristics of included literature.

Author, year	Clinical trial number	Phase	Country	Experimental arm	Control arm	Experimental number	Control number	Treatment line	Blind
Antoniotti (2022) (41)	NCT03721653	II	Italy	Atezolizumab + FOLFOXIRI + Bevacizumab	FOLFOXIRI + Bevacizumab	68	134	1	Open-label
Lenz (2024) (42)	NCT03414983	II/III	Multi-countries	Nivolumab + mFOLFOX6 ± Bevacizumab	mFOLFOX6 ± Bevacizumab	127	68	1	Open-label
Mettu (2022) (46)	NCT02873195	II	United States	Atezolizumab + Capecitabine + Bevacizumab	Capecitabine + Bevacizumab	87	46	≥2	Open-label
Ree (2024) (43)	NCT03388190	II	Norway	Nivolumab + FLOX	FLOX	38	38	1	Open-label
Tabernero (2022) (45)	NCT02291289	II	Multi-countries	Atezolizumab + FP + Bevacizumab	FP + Bevacizumab	148	297	1	Open-label
Wang (2024) (44)	NCT04547166	II	China	Serplulimab + XELOX + HLX04	Placebo + XELOX + Bevacizumab	57	57	1	Double-blind

FOLFOXIRI, fluorouracil, leucovorin, oxaliplatin, irinotecan; mFOLFOX6, oxaliplatin, leucovorin, fluorouracil; FLOX, fluorouracil, leucovorin, oxaliplatin; FP, fluorouracil, leucovorin; HLX04, a Bevacizumab biosimilar that functions as an anti-vascular endothelial growth factor monoclonal antibody; XELOX, oxaliplatin, capecitabine.

### 3.3 Quality assessment of included studies

The Cochrane Risk of Bias tool was used to assess the methodological quality of the six included studies. Regarding random sequence generation, five studies clearly described the randomization method and were rated as low risk; one study did not provide a clear description and was rated as unclear risk. For allocation concealment, five studies implemented adequate measures and were judged to have low risk, while one study did not clearly report the allocation concealment process, resulting in an unclear risk rating. In terms of blinding of participants and personnel, five studies adopted an open-label design and were judged to have high risk because of the lack of blinding, whereas one study was rated as low risk. For blinding of outcome assessment, four studies implemented blinding of outcome assessors and were rated as low risk; the remaining two studies had some risk of bias. Regarding completeness of outcome data, four studies reported complete outcome data and were rated as low risk; the other two studies did not clearly describe how attrition was handled, resulting in some risk of bias. All six studies reported the prespecified outcome measures with no evidence of selective reporting bias, and were rated as low risk. Details are provided in **Supplementary Figures 1** and **2**.

### 3.4 Outcome analysis

Pooled analyses of PFS and OS were performed for the five studies. The results showed that the experimental group achieved favorable PFS (HR = 0.84, 95%CI [0.73, 0.95], P = 0.007) with low heterogeneity (I²=0%) using a random-effects model (**Figure 2**). Moreover, when analyzed using a fixed-effects model, the results were identical (HR = 0.84, 95%CI [0.73, 0.95], P = 0.007). The experimental group also had longer OS (HR = 0.83, 95%CI [0.73, 0.94], P = 0.004) with low heterogeneity (I²=0%) using a random-effects model (**Figure 3**). In addition, when analyzed using a fixed-effects model, the results were identical (HR = 0.83, 95%CI [0.73, 0.94], P = 0.004).

**Figure 2:**
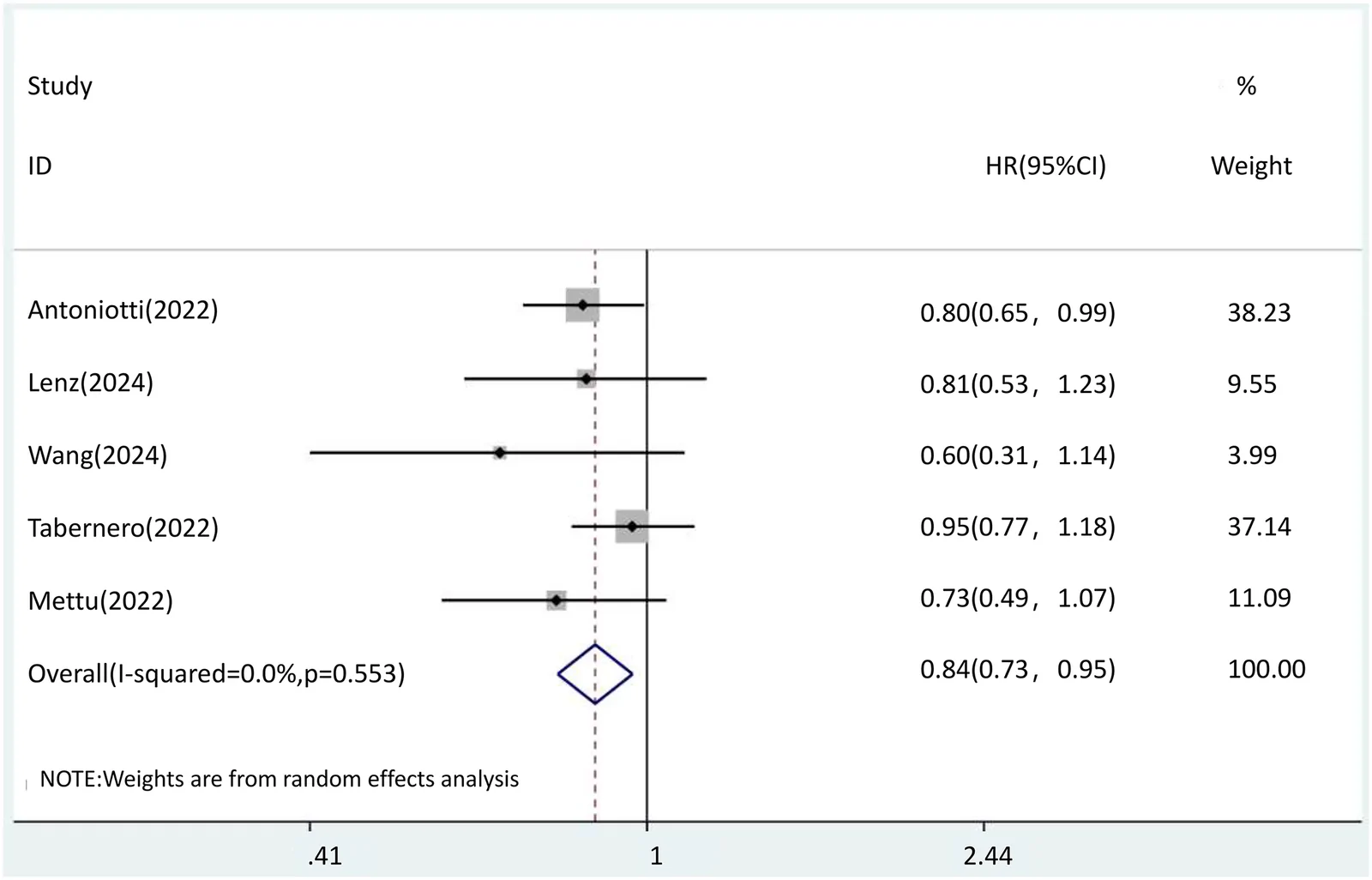
Forest plot of PFS for PD-1/PD-L1 inhibitors combined with chemotherapy with or without anti VEGF in pMMR mCRC (p=0.007).

**Figure 3:**
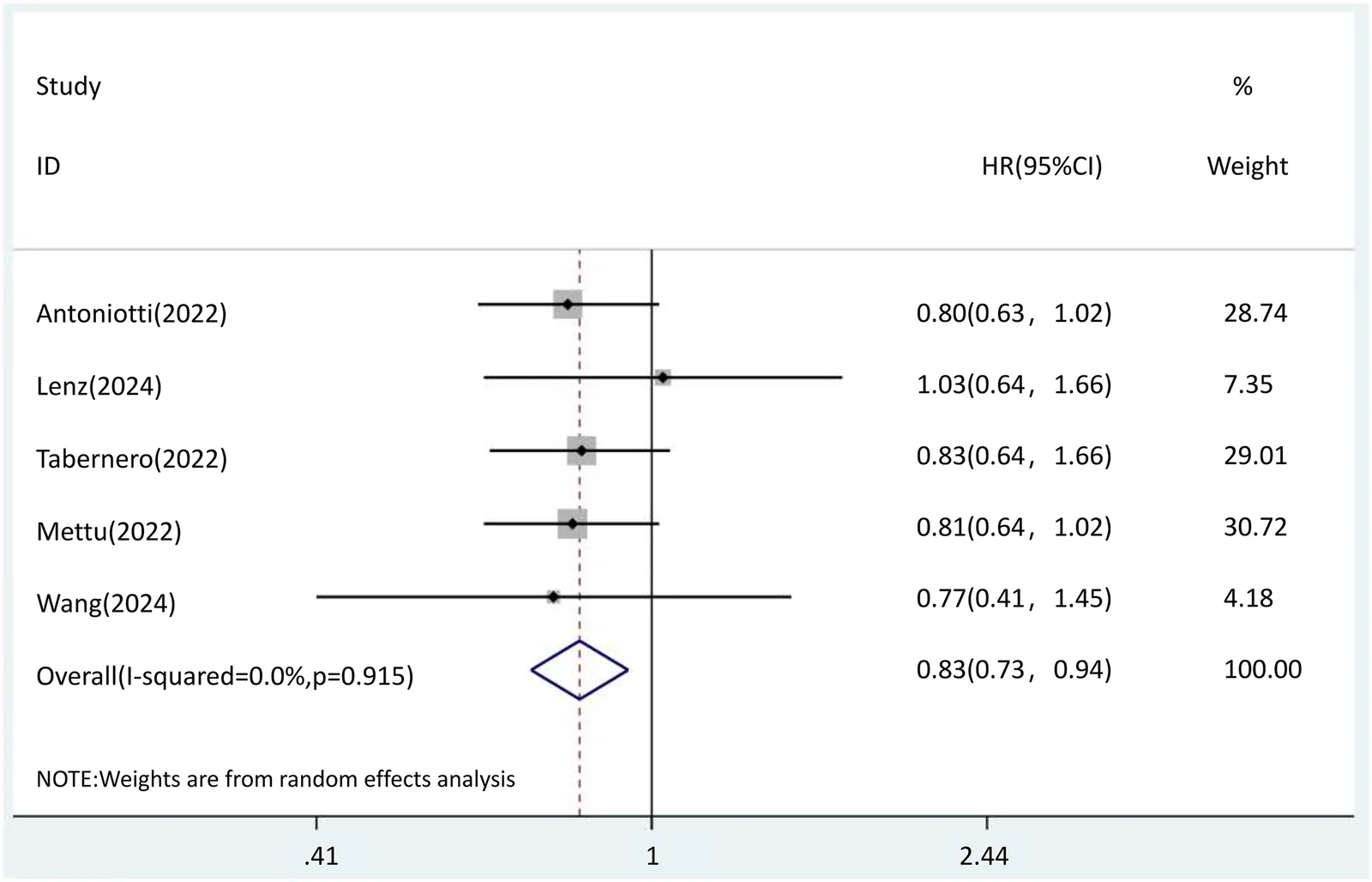
Forest plot of OS for PD-1/PD-L1 inhibitors combined with chemotherapy with or without anti VEGF in pMMR mCRC (p=0.004).

The certainty of evidence for the primary outcomes was assessed using the GRADE approach. For both OS and PFS, the certainty of evidence started at a high level and was downgraded by 2 levels to low because the risk of bias was very serious, as most included studies did not blind participants and investigators. See **Supplementary Table 2** for details.

### 3.5 Subgroup analysis

To explore sources of heterogeneity, subgroup analyses of PFS were conducted. Among male participants, the experimental group showed better PFS than the control group (HR = 0.77, 95%CI [0.64, 0.94], P = 0.010); however, no significant difference was observed in female participants (HR = 0.88, P = 0.515). According to age subgroups (<60–65 years vs. ≥60–65 years), neither younger participants (HR = 0.83, P = 0.104) nor older participants (HR = 0.85, P = 0.221) showed a significant difference in PFS between the experimental and control groups. Among participants with good performance status (ECOG = 0), the experimental group had better PFS than the control group (HR = 0.73, 95%CI [0.56, 0.95], P = 0.018); among those with poorer performance status (ECOG = 1/2), no difference was found (HR = 1.04, P = 0.757). Among participants with liver metastases, the experimental group demonstrated superior PFS compared to the control group (HR = 0.82, 95%CI [0.68, 0.98], P = 0.027); whereas in those without liver metastases, no significant difference was observed (HR = 0.84, P = 0.301). Regardless of left-sided (HR = 0.85, P = 0.142) or right-sided (HR = 0.74, P = 0.081) tumor location, there was no significant difference between the experimental and control groups. Among participants who had received prior adjuvant therapy, no significant difference was observed (HR = 1.20, P = 0.671); however, among those without prior adjuvant therapy, the experimental group was superior (HR = 0.81, 95%CI [0.67, 0.98], P = 0.030). For PD-L1 combined positive score (CPS) ≥ 1, there was no significant difference between the experimental and control groups (HR = 0.78, P = 0.251),whereas for PD-L1 CPS < 1, PFS was longer in the experimental group (HR = 0.53, 95%CI [0.30, 0.95], P = 0.032). Among participants with synchronous metastases, the experimental group had better PFS than the control group (HR = 0.79, 95%CI [0.63, 0.99], P = 0.037); among participants with metachronous metastases, no difference was observed (HR = 1.44, P = 0.156). Whether participants had a single metastatic lesion (HR = 0.95, P = 0.742) or multiple lesions (HR = 0.83, P = 0.163), there was no difference between the two groups. In participants with Kirsten rat sarcoma viral oncogene homolog (KRAS) wild-type status, no significant difference was observed between the experimental and control groups (HR = 0.91, P = 0.733); however, in those with KRAS mutations, the experimental group had better PFS than the control group (HR = 0.49, 95%CI [0.30, 0.80], P = 0.005). The results of the subgroup analyses are presented in **Table 2**.

**Table 2:** Subgroup analysis of progression-free-survival.

Subgroup	No. of studies	HR	95%CI	P	Heterogeneity	
					I^2^	P
Sex						
Male	4	0.77	0.64, 0.94	0.010	0%	0.680
Female	4	0.88	0.59, 1.30	0.515	35.3%	0.201
Age						
<60-65	4	0.83	0.66, 1.04	0.104	0%	0.888
≥60-65	4	0.85	0.65, 1.11	0.221	0%	0.569
ECOG status						
0	4	0.73	0.56, 0.95	0.018	18.3%	0.299
1/2	4	1.04	0.79, 1.38	0.757	0%	0.633
Liver metastases						
Yes	5	0.82	0.68, 0.98	0.027	0%	0.845
No	5	0.84	0.60, 1.17	0.301	0%	0.598
Tumor location						
Left	4	0.85	0.69, 1.06	0.142	0%	0.479
Right	4	0.74	0.53, 1.04	0.081	0%	0.778
Previous adjuvant therapy						
Yes	2	1.20	0.51, 2.83	0.671	10.0%	0.292
No	3	0.81	0.67, 0.98	0.030	0%	0.862
PD-L1 CPS						
≥1	2	0.78	0.51, 1.19	0.251	0%	0.854
<1	2	0.53	0.30, 0.95	0.032	0%	0.460
Time to metastases						
Synchronous	2	0.79	0.63, 0.99	0.037	0%	0.958
Metachronous	2	1.44	0.87, 2.40	0.156	0%	0.475
Number of metastatic sites						
Single	2	0.95	0.69, 1.30	0.742	0%	0.711
Multiple	2	0.83	0.63, 1.08	0.163	0%	0.541
KRAS status						
Wild type	2	0.91	0.54, 1.54	0.733	0%	0.398
Mutant type	2	0.49	0.30, 0.80	0.005	0%	0.572

HR, hazard ratio; CI, confidence interval; ECOG status, Eastern Cooperative Oncology Group performance status; PD-L1, programmed death-ligand 1; CPS, combined positive score; KRAS, Kirsten Rat Sarcoma Viral Oncogene Homolog.

### 3.6 Publication bias and sensitivity analysis

Egger’s test was used to assess publication bias. A publication bias test was performed for PFS (P = 0.182), and no significant publication bias was observed. Sensitivity analysis for PFS was performed by sequentially excluding each study, and the pooled HR for PFS ranged from 0.64 to 0.98, indicating that the pooled results were stable. Detailed results are presented in **Supplementary Figure 3**. Subsequently, a publication bias test was performed for OS (P = 0.467). After excluding any single study, the pooled HR for OS ranged from 0.69 to 0.96, indicating stable results, as detailed in **Supplementary Figure 4**.

## 4 Discussion

In this meta-analysis of 6 RCTs comprising 1,165 patients with pMMR mCRC, the addition of PD-1/PD-L1 inhibitors to chemotherapy, with or without anti-VEGF agents, was associated with statistically significant improvements in both PFS and OS. The following discussion addresses the potential biological mechanisms underlying the efficacy of combination therapy and interprets the findings of the exploratory subgroup analyses.

Combination therapy first enhances the immune system’s ability to recognize tumors by increasing the immunogenicity of tumor cells and the level of antigen exposure. On the one hand, chemotherapy can induce immunogenic cell death (ICD). On the other hand, chemotherapy can enhance tumor antigenicity and broaden the spectrum of antigen presentation. Furthermore, chemotherapy can induce the release of tumor-associated antigens (TAAs) (47). Patients without prior adjuvant therapy may derive greater benefit from this mechanistic rationale. Prior chemotherapy can lead to immune exhaustion, whereas treatment-naive patients retain more intact T-cell functionality and diversity, thereby providing a basis for the reactivation of antitumor immune responses (48, 49). Furthermore, these patients generally have better performance status (lower ECOG scores), and together, these factors maximize the overall benefit of combination therapy.

Combination therapy promotes immune cell recruitment and infiltration by inducing chemokine expression and improving tumor vascular structure. First, chemotherapeutic agents can induce tumor cells to produce multiple chemokines, thereby enhancing the ability of T cells to migrate to the tumor site (50). Second, anti-VEGF therapy can improve the physical conditions for immune cell entry into the tumor through vascular normalization (51). The pMMR tumors typically exhibit abnormal vascular networks with barriers that are difficult for cells and drugs to penetrate (52). Anti-VEGF agents inhibit the VEGF/VEGFR signaling pathway, promote vessel maturation, and reduce interstitial fluid pressure, thereby facilitating the infiltration of effector T cells into the tumor parenchyma (53). In liver metastases, VEGF-driven vascular abnormalities and immunosuppression may be particularly prominent. Therefore, anti-VEGF therapy not only improves vascular perfusion and drug delivery but may also reverse the immune-exclusion status of liver metastases through dual mechanisms of “vascular normalization and immune sensitization” (54, 55). Therefore, in our study, the presence of liver metastases appeared to define a favorable subgroup in exploratory analyses.

Furthermore, after anti-VEGF therapy, some tumors upregulate chemokines associated with T-cell recruitment, such as CXCL9, CXCL10, and CXCL11. These chemokines can further attract CD8+ T cells into the tumor via the CXCR3 axis, shifting the tumor microenvironment from an immune-desert phenotype to an inflammatory infiltrated phenotype (56–59). The mechanistic efficacy of this approach may be particularly enhanced in patients with synchronous metastases. In synchronous metastases, CD8+ T-cell density is higher than in primary tumors, suggesting a pre-existing activated immune state locally, and combination therapy is more likely to unleash pre-existing antitumor immunity (60, 61). In contrast, metachronous metastases are enriched in FoxP3+ Tregs and exhibit high CD200 expression, indicating stronger immunosuppression and potentially less benefit (62). Therefore, among these two groups, patients with metachronous metastases may derive less benefit.

The exploratory subgroup analyses suggested a more pronounced PFS benefit in male than in female patients, although this between-subgroup difference was not formally tested. First, sex-related differences in tumor location may be an important factor. Previous studies have shown that CRC in males more frequently occurs in the left-sided or distal colorectum, whereas in females it more commonly occurs in the right-sided or proximal colon (63). Tumors arising from the right colon tend to be more aggressive, show suboptimal responses to conventional chemotherapy and some targeted agents, and confer an unfavorable prognosis (64, 65). Therefore, male patients may be more likely to benefit from chemotherapy and anti-VEGF therapy.

Patients with a favorable performance status (ECOG = 0) generally exhibit more competent host immunity, reduced systemic inflammation, and superior organ reserve. Consequently, they are better able to tolerate full-dose combination therapy and are more likely to achieve a sustained antitumor response mediated by chemotherapy, anti-angiogenic therapy, and PD-1/PD-L1 inhibitors (66). Notably, ECOG = 0 is also a favorable prognostic factor in mCRC (67).

In exploratory subgroup analyses, among patients receiving PD-1/PD-L1 inhibitors combined with chemotherapy and anti-VEGF therapy, those with KRAS mutations appeared to have better PFS than those with KRAS wild-type tumors. However, most previous studies suggest that KRAS mutations generally promote an immunosuppressive tumor microenvironment and may be associated with resistance to ICI monotherapy (68, 69). Given that this subgroup analysis was based solely on an indirect comparison of two studies and that our findings are inconsistent with some previous reports, this result should be regarded as an exploratory, hypothesis-generating observation that warrants prospective validation. Possible explanations include that chemotherapy combined with anti-VEGF therapy, which mediates vascular normalization, may partially remodel the immunosuppressive tumor microenvironment of KRAS-mutant tumors, restore T-cell infiltration, and thereby re-sensitize them to PD-1/PD-L1 blockade (70, 71). Alternatively, KRAS mutations may provide a molecular basis for combination immunotherapy through public neoantigens and MAPK-mediated PD-L1 stabilization (72–75). However, this interpretation remains speculative and should be treated with caution.

In pMMR mCRC, PD-L1 CPS is not a stable or reliable predictor of treatment benefit. A low baseline PD-L1 expression level does not guarantee persistently low expression after treatment, because chemotherapy and anti-VEGF agents can dynamically upregulate PD-L1 (76, 77). In the CheckMate-142 and KEYNOTE-016 studies, PD-L1 expression levels showed no significant association with ORR or survival outcomes after nivolumab or pembrolizumab therapy (7, 78). In our study, we observed a PFS benefit in patients with low CPS. Previous studies have reported that low PD-L1 expression may be associated with KRAS mutations; when KRAS mutations are present, PD-L1 expression is significantly decreased in both tumor cells and stromal cells, thereby affecting immune responses (79). This issue warrants further investigation, and the possibility of a statistical artifact cannot be excluded. All subgroup analyses were exploratory and hypothesis-generating in nature, and the resulting evidence should be interpreted with caution; in the absence of interaction tests, no definitive conclusion can be drawn regarding differential benefit between subgroups.

Finally, practical application requires assessing clinical benefit beyond statistical significance. In this meta-analysis, the pooled PFS HR was 0.84, corresponding to only a 16% relative reduction in progression or death risk, so absolute benefit is limited. Versus first-line chemotherapy plus bevacizumab in pMMR mCRC, adding PD-1/PD-L1 inhibitors has uncertain incremental value, plus immune-related toxicity and economic burden. Economic models show checkpoint inhibitors are not cost-effective even in dMMR/MSI-H mCRC, and less so in pMMR. Thus, despite a statistically significant PFS benefit, its clinical magnitude is small; adoption should be cautious, weighing limited benefit against toxicity and costs.

The main strength of this study lies in its comprehensive and systematic meta-analysis of the efficacy of PD-1/PD-L1 inhibitors combined with chemotherapy (with or without targeted agents) in the treatment of mCRC. Although previous meta-analyses have also reported on PD-1/PD-L1 inhibitors plus chemotherapy, they were limited by sample size and had insufficient statistical power for OS, failing to draw definitive conclusions regarding survival benefit. The present study adds two recently reported RCTs, thereby expanding the sample size and increasing statistical power, and consequently yielded a positive OS result. In addition, whereas previous meta-analyses performed subgroup analyses only by sex and ECOG performance status, this study conducted more comprehensive subgroup analyses based on clinical and molecular characteristics, including PD-L1 CPS, KRAS mutation status, presence or absence of liver metastasis, and prior adjuvant therapy.

However, this study has several limitations. First, the sample size was limited and the number of included RCTs was small. Most subgroup analyses were based on data from only a few studies, with limited statistical power, and no interaction tests were performed to assess differences between subgroups, nor were interaction P values reported. Therefore, the apparent differences observed between subgroups should be regarded as exploratory, hypothesis-generating signals rather than confirmed evidence of differential benefit. Second, although statistical heterogeneity was low for both PFS and OS (I²=0%), clinical heterogeneity remained among the included studies, including differences in lines of therapy, combination regimens, and control group design. In addition, because most included studies were open-label phase II or phase II/III clinical trials without blinding of participants and investigators, the certainty of evidence for both OS and PFS was rated as low in the GRADE assessment, primarily reflecting a high risk of bias. These findings should therefore be interpreted with caution and await confirmation by future adequately powered, large-scale phase III RCTs.

## 5 Conclusion

This meta-analysis indicates that combining PD-1/PD-L1 inhibitors with chemotherapy, with or without anti-VEGF agents, improves outcomes in patients with pMMR mCRC. Exploratory subgroup analyses suggested a trend toward a more pronounced PFS benefit in male patients, those with good performance status (ECOG = 0), presence of liver metastases, no prior adjuvant therapy, PD-L1 CPS < 1, synchronous metastases, and KRAS mutations; however, in the absence of interaction tests, these observations are hypothesis-generating and require prospective validation. Overall, these findings suggest that PD-1/PD-L1 inhibitors plus chemotherapy, with or without anti-VEGF therapy, could represent a potential first-line treatment option for pMMR mCRC. However, given that most of the included studies were phase II clinical trials, the OS data remain immature, and no pooled safety analysis was performed, these results should be considered exploratory and await further validation in future large-scale, multicenter, phase III RCTs.

## Data Availability

The original contributions presented in the study are included in the article/**Supplementary Material**. Further inquiries can be directed to the corresponding author.

## Supplementary material

Supplementary Table 1 Characteristics of the studies included in this meta-analysis.

Supplementary Table 2 GRADE Summary of findings: PD-1/PD-L1 inhibitors plus chemotherapy with or without targeted agents compared with chemotherapy with or without targeted agents for people with pMMR mCRC.

Supplementary Figure 1 Risk of bias assessment in included studies.

Supplementary Figure 2 Summary of risk of bias across studies.

Supplementary Figure 3 Funnel plot with Egger’s test (p=0.182) and sensitivity analysis for PFS.

Supplementary Figure 4 Funnel plot with Egger’s test (p=0.467) and sensitivity analysis for OS.
