# Supplementary material for: PD-1/PD-L1 inhibitors in the treatment of pMMR mCRC: a meta-analysis based on randomized controlled trials
Source: Front Oncol. 2026 Oct 21;16:1969514. doi: 10.3389/fonc.2026.1969514 (PMC13730918; doi:10.3389/fonc.2026.1969514)
Supplement: Supplementary file 1 [file Table1.docx]

**Supplementary Table 1. Characteristics of the studies included in this meta-analysis.**

| Author, year | Recruitment time | Gender (M/F) | Experimental-arm patients | | | | Control-arm patients | | | |
| --- | --- | --- | --- | --- | --- | --- | --- | --- | --- | --- |
|  |  |  | Median age, years (range) | ECOG PS, n  0/1/≥2 | Mismatch repair status, n pMMR/dMMR/missing date | Tumor location, n left/right or transverse/unknow | Median age, years (range) | ECOG PS, n  0/1/≥2 | Mismatch repair status, n pMMR/dMMR/missing date | Tumor location, n left/right or transverse/unknow |
| Antoniotti (2022) | 2018.11-2020.2 | 125/93 | 60.0 (52-67) | 125/20/0 | 134/8/3 | 80/65/0 | 61.0 (54-66) | 62/11/0 | 68/5/0 | 41/32/0 |
| Lenz (2024) | 2018.02-2019.04 | 119/76 | 58.0 (24-85) | 76/51/0 | 121/6/0 | 76/39/12 | 56.0 (24-78) | 41/27/0 | 61/7/0 | 39/20/9 |
| Mettu (2022) | 2017.09-2018.06 | 77/51 | 59.0 (53-66) | 42/40/0 | 69/9/4 | 59/23/0 | 55.5 (50-63) | 26/20/0 | 41/4/1 | 35/11/0 |
| Ree (2024) | 2018.05-2021.10 | 41/35 | 60.5 (43-80) | 23/15/0 | 38/0/0 | 27/11/0 | 65.0 (38-39) | 21/17/0 | 38/0/0 | 27/11/0 |
| Tabernero (2022) | 2015.08-2016.11 | 271/174 | 62.0 (25-87) | 173/119/5 | 247/5/45 | 207/56/34 | 62.0 (27-83) | 79/69/0 | 124/2/22 | 106/25/17 |
| Wang (2024) | 2021.07-2022.01 | 83/31 | 61.0 (25-74) | 12/44/0 | 40/4/13 | 39/18/0 | 58.0 (26-73) | 17/40/0 | 50/0/7 | 41/16/0 |

M, male; F, female; dMMR, deficient mismatch repair; pMMR, proficient mismatch repair; ECOG PS, Eastern Cooperative Oncology Group performance status.
