# Supplementary material for: PD-1/PD-L1 inhibitors in the treatment of pMMR mCRC: a meta-analysis based on randomized controlled trials
Source: Front Oncol. 2026 Oct 21;16:1969514. doi: 10.3389/fonc.2026.1969514 (PMC13730918; doi:10.3389/fonc.2026.1969514)
Supplement: Supplementary file 2 [file Table2.docx]

**Author(s):**

**Question:** PD-1/PD-L1+chemotherapy± targeted agents compared to chemotherapy± targeted agents for pMMR mCRC

**Setting:**

**Bibliography:**

| **Certainty assessment** | | | | | | | **№ of patients** | | **Effect** | | **Certainty** | **Importance** |
| --- | --- | --- | --- | --- | --- | --- | --- | --- | --- | --- | --- | --- |
| **№ of studies** | **Study design** | **Risk of bias** | **Inconsistency** | **Indirectness** | **Imprecision** | **Other considerations** | **PD-1/PD-L1+chemotherapy± targeted agents** | **chemotherapy± targeted agents** | **Relative (95% CI)** | **Absolute (95% CI)** |  |  |
| **overall survival (assessed with: HR)** | | | | | | | | | | | | |
| 5 | randomised trials | very serious^a^ | not serious | not serious | not serious | none | 487/1089 (44.7%) | 602/1089 (55.3%) | **HR 0.83** (0.73 to 0.94) | **66 fewer per 1,000** (from 109 fewer to 22 fewer) | ⨁⨁◯◯ Low^a^ | CRITICAL |
| **progression-free survival (assessed with: HR)** | | | | | | | | | | | | |
| 5 | randomised trials | very serious^a^ | not serious | not serious | not serious | none | 487/1089 (44.7%) | 602/1089 (55.3%) | **HR 0.84** (0.73 to 0.95) | **61 fewer per 1,000** (from 109 fewer to 18 fewer) | ⨁⨁◯◯ Low^a^ | CRITICAL |

**CI:** confidence interval; **HR:** hazard ratio

#### Explanations

a. Blinding of participants and personnel was not performed in most studies.
